# Shifting Paradigms: The Case of Autologous Reconstitution after an Upfront Matched Unrelated Hematopoietic Cell Transplantation for Severe Acquired Aplastic Anemia in a Child

**DOI:** 10.3390/medicina59111890

**Published:** 2023-10-24

**Authors:** Cécile Pochon, Marion Lubnau, Simona Pagliuca

**Affiliations:** 1 Pediatric Onco-Hematology Department, Nancy University Hospital, 54500 Vandoeuvre-lès-Nancy, France; m.lubnau@chru-nancy.fr; 2 CNRS UMR 7365, IMoPA, Biopole of University of Lorraine, 54500 Vandoeuvre-lès-Nancy, France; 3 Hematology Department, Nancy University Hospital, 54500 Vandoeuvre-lès-Nancy, France

**Keywords:** severe aplastic anemia, MUD HCT, graft failure, autologous reconstitution

## Abstract

During the last few years, the therapeutic landscape of idiopathic aplastic anemia (IAA) has been profoundly revolutionized by the increased use of alternative transplant procedures, such that today hematopoietic cell transplantation (HCT) from a matched unrelated donor (MUD) has been suggested as a possible first line strategy in pediatric patients with severe IAA, in the absence of a matched related donor. However, in this particular context, outcomes and early and long-term toxicities remain to be determined, as compared to non-transplant procedures. While prospective trials are ongoing, we report here the case of a 12-year-old boy with IAA, receiving an upfront bone marrow HCT from a MUD, who experienced early graft rejection associated with autologous hematological recovery, which could induce remission of his hemopathy. This case offers the opportunity to discuss the challenges associated with these new transplant paradigms and provides a brief review of the literature regarding the issue of autologous recoveries after allogeneic HCT in IAA.

## 1. Introduction

Few moments in the story of a rare disease may represent a breakthrough translating into changes in clinical practices. For idiopathic aplastic anemia (IAA) and paroxysmal nocturnal hemoglobinuria (PNH) this is happening now. The demonstrated advantage of the triple immunosuppressive therapy based on the results of the recently published RACE trial [1], the extensive pipeline of anti-complement agents for PNH patients [2,3,4,5], the upfront use of matched unrelated (MUD) or other alternative donors [6,7,8,9,10,11] for allogeneic hematopoietic cell transplant (allo-HCT) indications, are unprecedentedly revolutionizing the field. These changes in paradigms may give rise to challenging scenarios. We report here the case of a 12-year-old patient receiving a bone marrow allograft from a MUD who presented with early rejection followed by autologous reconstitution. Through this case, we take the opportunity to discuss the challenges related to the new transplant algorithms in pediatric IAA, providing a brief review of the literature on the issue of autologous hematopoietic recovery after allogeneic HCT procedures in this hematological disorder.

## 2. Case Report

We report here the case of a 12-year-old boy diagnosed with severe IAA and followed at our institution. He did not have any medical or surgical history. At diagnosis, peripheral blood counts showed the following values: hemoglobin (Hb) 4.2 g/dL, reticulocytes 14 × 10^9^/L, mean corpuscular volume 80 fl, neutrophils 0.4 × 10^9^/L and platelets 14 × 10^9^/L. Bone marrow cytology confirmed poor cellularity, absence of dysplasia and blasts, orienting toward a bone marrow failure (BMF) disorder, while karyotype and fluorescence in situ hybridization analysis were normal. PNH clonality was not observed. Born at term, his birth weight was 3030 g. Although morphologically there was no evidence for an inherited disorder, fetal Hb was elevated and the chromosomal breakage test was doubtful. The patient was an only child but had numerous human leukocyte antigen (HLA) 10 out of 10 MUDs on the international marrow donor registry. Given the severity of the disease and the initial suspicion of inherited BMF, we validated the indication of a bone marrow MUD transplant, as an urgent first line treatment. Finally, the full inherited BMF work-up turned out to be negative: normal *FANCD2* test, normal chromosome breakage test, normal telomere length, confirming the acquired nature of this BMF.

Before this diagnostic ascertainment, the patient received a bone marrow graft from his MUD (a 23-year-old man) as planned, with a conditioning regimen based on fludarabine (30 mg/m^2^/day from day 7 to day 3), cyclophosphamide (60 mg/kg/day from day 3 to day 2), and alemtuzumab (0.3 mg/kg/d from day 6 to day 4), FCC [12]. Graft cell richness was satisfactory (4.93 × 10^8^ total nucleated cells/kg, including 5 × 10^6^ CD34/kg), and donor/recipient characteristics were: cytomegalovirus (CMV) serostatus −/−, Epstein–Barr virus (EBV) serostatus −/− toxoplasma gondii serostatus −/−, ABO group O+/AB+. Graft versus host disease (GVHD) prophylaxis included ciclosporin alone, started on day 1. The conditioning regimen was very well tolerated without extra-hematological toxicities, nor virus replication or other documented infections. He experienced one episode of febrile neutropenia without microbiological documentation, amended with empirical broad-spectrum antibiotics. He further developed fever and erythematous maculopapular rash during the engraftment phase, which led to the diagnosis of an engraftment syndrome (ES). Corticosteroid therapy was thus initiated at 1 mg/kg/day from day 12. Full neutrophil recovery occurred at day +19 while no platelet reconstitution was observed. A bone marrow exam, performed at day +24, showed a reduced cellularity with absence of megakaryocytes and a hypoplastic granular lineage with a 97% donor chimerism, confirmed in peripheral blood at day +30. Corticosteroid therapy was gradually withdrawn. Ciclosporin levels were maintained in a range of 200–250 µg/L.

**Figure 1:**
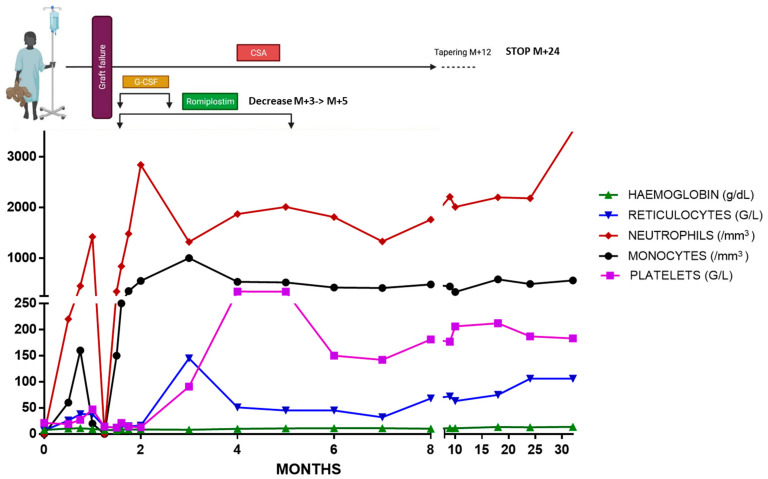
Patient clinical course after BMT. Abbreviations: G-CSF: granulocytic stem cell factor; HCT: hematopoietic cell transplantation. Figure created with BioRender.com.

Unfortunately, abrupt pancytopenia appeared at day +35 after transplant (leukocytes 0.06 × 10^9^/L, platelets 6 × 10^9^/L, Hb 8.4 g/dL and reticulocytes 14 × 10^9^/L) without evidence of bacterial, fungal or viral infection (negative EBV, CMV, adenovirus, human herpes virus 6 (HHV6) and parvovirus B19 DNAemias). A bone marrow smear was acellular, showing only eosinophil and mastocyte infiltration. Chimerism analysis at day +37 showed 27% and 16% of donor cells in bone marrow and peripheral blood, respectively, indicating early secondary graft rejection. After stimulation with granulocyte-colony stimulating factor (G-CSF), neutrophils increased progressively. Despite resumption of corticosteroid therapy at 1 mg/kg/day and continuation of ciclosporin, blood chimerism at day +47 showed only 3% of donor cells. In view of the progressive autologous reconstitution of the granulocyte lineage following administration of G-CSF, we decided to start a weekly romiplostim treatment with increasing doses up to 10 µg/kg. Cyclosporine was continued at a level range of 150–200 µg/L. Corticosteroid therapy was discontinued. The patient showed progressive autologous hematological reconstitution at day 60 with reticulocytes > 50 × 10^9^/L, platelets > 20 × 10^9^/L and neutrophils > 0.5 × 10^9^/L despite discontinuation of G-CSF, without transfusion needs. At 3 months post-transplant we observed a full platelet recovery that prompted a gradual reduction in the thrombopoietin receptor agonist, with discontinuation at 5 months after allo-HCT. Ciclosporin was maintained at an effective dose until 1 year after transplant and gradually reduced, with definitive discontinuation at 2 years. There were no notable infections or specific drug toxicities (apart from those expected, i.e., hypertrichosis induced by cyclosporine) during the patient’s close medical follow-up. Up to date at 2 years and 8 months after the transplant procedure, the patient is in complete remission of his BMF, with autologous reconstitution and without any immunosuppressive regimen (Figure 1).

## 3. Discussion

We report here the proof of concept that, although graft failure may impair the success of allo-HCT procedures, especially following non-matched related donor transplants, the possibility of autologous hematopoietic reconstitution, with durable blood count normalization, should be taken into account, before considering further salvage therapies including a second allo-HCT. This approach could be discussed in particular in the case of primary rejection of the first-line alloHCT, which includes in vivo T cell depletion, in patients who have not received any immunosuppressive treatment prior to transplantation. In view of the risks associated with prolonging severe aplastic anemia, arguments such as a response to G-CSF to limit the risk of infection, the absence of a threatening active infection or major hemorrhagic risk, and a correct transfusion yield, could support a wait-and-see strategy with the addition of a TPO analogue treatment, combined with the maintenance of calcineurin inhibitors, before organizing a 2nd allograft in the event of failure. However, insofar as this illustration reflects the evolution of a single patient, the message of this work must be limited by the impossibility of generalizing this medical history to all patients. Larger scale studies are needed to support this recommendation.

Considering the risks of failure, relapse, clonal evolution or ciclosporin dependence after immunosuppressive treatment with long-term event free survival (EFS) estimated at 10–30% [13,14] and given the promising survival outcomes shown by upfront MUD HCT procedures in pediatric IAA patients, with EFS estimated at 95% at 5 years, this last strategy was suggested as a possible first line treatment in this setting [15]. A series of 29 children in the United Kingdom with IAA undergoing MUD bone marrow transplant as first-line treatment demonstrated the superiority of this approach as compared to immunosuppressive treatment in the pediatric population, and the equivalence of these transplants in comparison with sibling donor-based procedures [14]. The conditioning regimen used in this experience was FCC. In this series one child developed a primary graft failure and was rescued by a second transplant from a different donor. A pilot study in North America demonstrated the feasibility of this approach in 10 patients, who received a conditioning regimen with fludarabine 120 mg/m^2^, rabbit anti-thymoglobulin (ATG) 9 mg/kg, cyclophosphamide 50 mg/kg, and total body irradiation (TBI) in a single dose of two grays, showing excellent outcomes [16]. Here early rejection was observed in one patient, leading to a second transplant procedure from another donor, with a favorable outcome.

In a retrospective series of 56 children receiving sibling or MUD allografts, after a conditioning regimen with fludarabine, cyclophosphamide and ATG, including 32 in the first line, none developed graft rejection [17]. In another study, including 50 patients (children and adults) who received HCT between 1999 and 2009 conditioned with FCC regimen, rejection occurred in six patients, of whom four transplanted from an MUD. Autologous recovery, as in our case, was seen in two out of these six patients while two received a 2nd allograft and two died in graft failure [18]. A study from the Severe Aplastic Anemia working party (SAAWP) of the European Group for Blood and Marrow Transplantation (EBMT) showed that of 38 patients transplanted in the second line with alternative donors (unrelated matched or mismatched or haploidentical), seven (18%) experienced a graft failure [19]. Of them, three3 eventually underwent autologous reconstitution.

A clinical trial is currently underway in France (NCT05419843) to report on the feasibility and efficacy of first-line MUD transplant procedures in patients < 18 years. Preparative regimens proposed to include fludarabine 120 mg/m^2^, cyclophosphamide 120 mg/kg, and ATG 15 mg/kg for children under 14 years of age, versus ATG 7.5 mg/kg and TBI 2 grays for children over 14 years of age.

If graft rejection has been largely described in the literature, the epidemiology of autologous recovery following allo-HCT in BMF is less known, although this has been better documented in matched related transplants. The first description of autologous reconstitution after allograft transplantation dates back to 1976 [20]. In a retrospective study by the SAAWP of the EBMT, carried out between 1973 and 2005, 45 cases of autologous reconstitution out of 1205 patients were identified in the registry, giving an estimated cumulative incidence of 4.2%. Five patients subsequently relapsed with the initial BMF. In this study the type of donor did not affect the incidence of autologous reconstitution, and 12 patients had autologous reconstitution after a MUD transplant. The 10-year overall survival rate for these patients was 84%. A total of seven patients died (from infections or relapse of IAA). A prolonged time between transplantation and diagnosis, and conditioning with cyclophosphamide and anti-thymoglobulin, were factors associated with graft rejection and autologous reconstitution [21]. The low incidence of autologous reconstitution in this study could be explained by the characteristics of this historical cohort, with the majority of transplant procedures carried out after failure of one, or 2, immunosuppressive treatments. The group reported an increasing incidence of this event after 1991, possibly due to improvement of chimerism analysis techniques and therefore a greater ability to detect autologous reconstitution. In another Brazilian study, involving 104 patients, mainly transplanted with bone marrow from a sibling donor as first-line treatment for severe IAA, autologous reconstitution was defined as less than 50% chimerism more than 18 months after transplant. The rate of autologous reconstitution was estimated at no less than 36% in the group of patients who received cyclophosphamide conditioning alone. It should be noted that in this study there was no in vivo T depletion by ATG. This team raised the long-term problem of the risk of clonal transformation of hematopoietic cells in the event of autologous reconstitution, but did not report any incidence of hematological malignancy in their cohort [22]. Finally, cases of autologous reconstitution with good long-term survival have been described following cord blood transplantation as 1st-line or further treatment for IAA [23,24,25]. The excellent survival outcomes of patients with autologous reconstitution presented in Piccin et al. study, and our case, prompt further reflections in terms of the management of this particular situation, especially in the era of “MUD upfront”.

Based on recent advancements in preparative regimens and transplant procedures, as a breakthrough in the treatment algorithm of IAA, MUD allo-HCT is shifting its place from second-line to first-line treatment in younger patients, with promising results in terms of survival and incidence of complications in early studies. However, large-scale prospective trials and long-term analyses are need to confirm the superiority of this procedure on intensive immunosuppression. Primary or secondary graft failure remains a well-established issue after second-line or alternative-donor transplant procedures in BMF and management of this complication remains an unmet need, with a second allograft often considered as a salvage strategy. Our case illustrates how long-lasting autologous recoveries after graft failure can sometimes occur inducing a state of complete remission, formally similar to responses seen after intensive immune suppression. The incidence of this event remains to be determined after first-line MUD transplant procedures in future prospective studies.

## Data Availability

Not applicable.
